# The American Academy of Dental Surgery

**Published:** 1877-02

**Authors:** 


### ARTICLE II.

### *The American Academy of Dental Surgery.*

[Reported for the American Journal of Dental Science.]

A regular meeting of the Academy was held on Tuesday evening, December 16th. President, Dr. Geo. H. Perine, in the chair. The Corresponding Secretary laid before the Academy, important correspondence from members of the Medical profession, as to the best means of forming a closer union between the medical and dental professions. Presentations were made to the library and museum, and a vote of thanks returned. An interesting collection of dental curiosities were exhibited, consisting of antique dental and surgical instruments, artificial teeth, also a large fossil of the lower maxillary, and a tooth of a Mammoth discovered in Nottingham, England. While excavating through sand-stone rock, a laborer came upon a stone like substance which proved to be a tooth, the centre of the stump being greatly worn by constant mastication, but the enamel lines of the series of molars being bright and clear. The tooth was found in the sand-drift eight feet six inches below the surface, and its weight is four and a half pounds. An india-rubber tongue, made in Paris, for a patient who had lost this organ from cancer, was also exhibited, together with photographs of various kinds of instruments, found in the ruins of Pompeii; also, a copy of a painting, representing a surgical operation, which gave rise to an interesting discussion.

After some pertinent remarks on the working of the Academy, the President announced with sorrow and regret, that since the last meeting, we have been called upon to part with one of their most esteemed Fellows, Dr. A. McElroy. Resolutions were presented and adopted condoling with the relatives and friends of the deceased.

The preliminary business having been gone through with, the following paper by C. P. Hunt, on Neuralgia, was read:

As we shall find it most convenient to describe the various forms of neuralgia in their automical, rather than in their physiological relations, this will be the proper place to treat one of the most common and painful varieties—*prosopalgia*, or as it is generally termed *tic doulereux*. This is, for the most part an affection of the trigeminus, or fifth pair of nerves, but in asmuchas the *portio dura* after passing through the parotid gland, is connected with a twig of the trigeminus, the pain is sometimes, though rarely felt also in the course of that nerve, commonly but one branch of the trigeminus, the superior maxillary of one side is affected, but not unfrequently two, and sometimes all three of the branches are involved, and the pain by implicating the opposite branches may even extend to the other side of the face.

As already stated, the most frequent form of prosopalgia is that involving the middle branch of the trigeminus. The pain is generally first felt in or near the infra-orbital foramen, and being seated in the nerve of that name, extends to the inner canthus of the eye, the lower eyelids, the muscles about zygoma, those of the cheek especially the buccinator, the upper lip and the alae of the nose. Subsequently the trunk of the nerve and the branches given off from it in its passage through the infra-orbital canal become affected, the pain being felt in the palate, tongue, zygomatic fossa, the upper teeth and the nasal cavity. As the disease progresses, the pain may extend as in other forms of *prosopalgia* to all parts of the face. The pain is never continual but occurs in paroxysms of greater or less violence and duration; when fully formed the paroxysms are frequently attended by a copious salivation.

Next in order of frequency, the pain commences near the supraorbital foramen and extending outward along the branches of the frontal nerve and its ramifications, is experienced in the soft parts covering the anterior of the cranium or it may extend in the opposite direction along the trunk of the nerve, and be felt at the bottom of the orbit. Subsequently the *tunica conjunctiva* and adjacent parts become affected producing redness of the conjunctiva and lids with more or less lachrymation and swelling. Sometimes it causes extreme photophobia, the eye becoming so exceedingly painful and sensitive to light, that the patient can scarcely tolerate a single ray. Finally, as in other cases the pain passes beyond the parts supplied by the frontal nerve, extending itself to the supraorbital, the maxillary and sometimes through communicative filaments, to the facial, temporal and occipital nerves.

Less common than either of the preceding, but when confirmed an equally intense and obstinate form of *prosopalgia* is that affecting the inferior maxillary nerve. The pain is generally first felt at or near the anterior mental foramen, and extends to the teeth, lower lip, chin, temple and neck. As in the preceding forms of the disorder, the associated branches of the trigeminus, as well as the *portio dura* of the seventh, frequently become implicated, and then the paroxysms of pain, become more or less general over one side of the face and head. When the motor nerves become implicated, the muscles supplied by them twitch convulsively, producing distortion of the features and in some cases more or less spasmodic action of more distant parts, the latter being caused doubtless by the extreme pain. The irritability of the affected nerves frequently become so great during the paroxysms, that the impressions produced merely by movement, as in talking, sneezing and in chewing, or by currents of cold air, etc., are often sufficient to renew the attacks. The pains, are of a shooting, rending or burning character, and when the paroxysm is at its height, they frequently become so intolerable, that the patient is utterly unable to suppress his cries.

*Prosopalgia*, is very liable with hemicrania and rheumatism. From the former it may be distinguished by the seat of the pain, corresponding accurately with the course and distribution of the affected nerves, and from the latter, by the exacerbation being provoked by the slightest touch, by the limited duration of the paroxysm, and by the intolerable character of the pain. From ordinary toothache it may be distinguished by the transient character and rapid succession of the pains, the convulsive twitchings of the muscles and the coursing of the pain along the tracks of the affected nerves. With reference to those cases in which the *portio dura* of the seventh pair of nerves becomes implicated they are sometimes exceedingly difficult to distinguish from those in which only the branches of the trigeminus are invo,[ved the chief of the trigeminus, but in consequence of its communication with the other nerves of the face the agony soon becomes general over the entire side of the head.

The causes of this affection are generally very uncertain and obscure, at times, particularly when the main trunk is affected. It can then be traced to tumors, or bony growths pressing upon the affected nerves, and occasionally the attack can be satisfactorily referred to such causes as wounds, decayed teeth, the suppression of accustomed discharges, rheumatism, gout, syphilis, poisonous cosmetics, etc., but in the majority of cases, no known cause can be assigned. Even the most careful anatomical and pathological investigations generally fail elicit any satisfactory explanation. True to the affected nerves are sometimes found red and inflamed, but the ordinary absence of fever, the sudden transient and intermittent character of the attacks, their frequent occurrence in debilitated states of the system, and the usual absence of tenderness on pressure, are sufficient proofs that the cause, whatever it may be, is not always of an inflamatory character; probably the most frequent exciting cause is cold. Next to this, those causes which induce cephalgia, such as mental emotion, severe mental and physical labor, excess in eating and drinking, the abuse of spirituous liquors, tea, coffee and tobacco, excessive venery, etc., no doubt contribute greatly to produce it in those who are predisposed to the affection. What particular class of persons are predisposed to it however, is not so clear; it is doubtful whether sex has any special influence in this direction, as some suppose, though there are peculiarities in the female constitution which undoubtedly predispose it to other forms of neuralgia, especially such as have their origin in the spine; probably what is called the nervous temperament, or an excitable disposition, furnishes as strong a predisposing cause as any, of which we have knowledge. The possibility, or even probability of a cure depends upon a variety of circumstances; when caused by malarious influences, general debility, pernicious habits, or by cold, a cure is generally easily effected; but when, on the other hand structural changes, such as tumors and other morbid growths give rise to it, there is but little hope of relief. Even in the milder forms of the disease, the patient frequently remains more or less subjected to the complaint as long he lives and death very seldom results from the attacks, however severe the paroxysms, but it always has a more or less pernicious effect upon the system, undermining the general health and rendering the mind feeble and the nervous system extremely sensitive and irritable. It follows from the purely subjective character and limited range of the symptoms, that the treatment of *prosopalgia* needs to be conducted with special reference to the cause. Hence it becomes necessary first of all to institute a careful scrutiny into the general state of the patient's health, his habits and surroundings, traveling as it were, beyond the boundaries of the symptomatic indications in order to ascertain if possible the true cause of the malady. In this way the prescriber is enabled to make his anatomical, physiological and pathological knowledge contribute not only to the diagnosis, but in a large proportion of cases to the cure of this obscure, obstinate and very painful disease; even with all the light which can be thrown upon it in this manner, the practitioner will often have great difficulty in selecting a suitable remedy, and will as frequently be disappointed; but it is evident that in no other way in many cases can there be any reasonable hope of success. Thus directed however, the symptomatic indications are generally sufficiently definite to suggest the proper remedy.

The subject was freely discussed by Drs. Perry, Johnston, Adams, Allen, Brown, Meigs, Hayward, James and Perine, when the meeting adjourned till the fourth Tuesday in January.

